# Pedicled lateral nasal wall flap for endoscopic skull base reconstruction: how I do it

**DOI:** 10.1007/s00701-026-06906-3

**Published:** 2026-05-16

**Authors:** Zhenhua Huang, Maohua Ding, Yue Ma, Xiaoguang Tong

**Affiliations:** 1 Department of Neurosurgery, Huanhu Hospital Affiliated to Tianjin Medical University, Tianjin, China; 2 Clinical College of Neurology, Neurosurgery and Neurorehabilitation, Tianjin Medical University, Tianjin, China; 3 Tianjin Key Laboratory of Cerebral Vascular and Neurodegenerative Diseases, Tianjin, China; 4 Tianjin Key Laboratory of Cerebral Blood Flow Reconstruction and Head and Neck Tumor New Technology Translation, Tianjin, China; 5 Huanhu Hospital, Tianjin University, Tianjin, China

**Keywords:** Lateral nasal wall flap, Nasoseptal flap, Inferior turbinate flap, Skull base reconstruction, Endoscopic endonasal surgery, Cerebrospinal fluid leak

## Abstract

**Background:**

The pedicled nasoseptal flap (Hadad-Bassagasteguy flap, HBF) has been considered as the workhorse for reconstruction of high-flow cerebrospinal fluid (CSF) leak following endoscopic endonasal skull base surgery. However, if the septal mucosa is affected by a tumor or the vascular pedicle is injured due to prior surgery, the use of HBF is precluded.

**Method:**

We illustrate the harvest techniques of the lateral nasal wall flap (LNWF) based on the posterior lateral nasal artery (PLNA).

**Conclusion:**

The LNWF is a robust intranasal regional flap with a consistent blood supply and comparable coverage surface, making it a reliable alternative when the HBF is unavailable.

**Supplementary Information:**

The online version contains supplementary material available at 10.1007/s00701-026-06906-3.

## Relevant surgical anatomy

The blood supply of the lateral nasal wall mainly comes from the facial artery anteriorly, the anterior ethmoidal artery superiorly, and the sphenopalatine artery (SPA) posteriorly. Following its entry into the nasal cavity, the SPA is divided into the nasal septal branch on the medial side and the turbinate branch on the lateral side. The latter, also known as the PLNA, contributes the main blood supply to the posterior pedicled LNWF [10].

PLNA originates from SPA near the sphenopalatine foramen, and in the descending course, it first branches the middle turbinate artery at the posterior edge of the middle turbinate, then it continues to descend submucosally posterior to the middle turbinate, reaching the posterior end of the inferior turbinate and giving off branches that distribute to the inferior turbinate and the inferior nasal meatus [7].

## Description of the technique

- 1. Make incisions

**Fig. 1:**
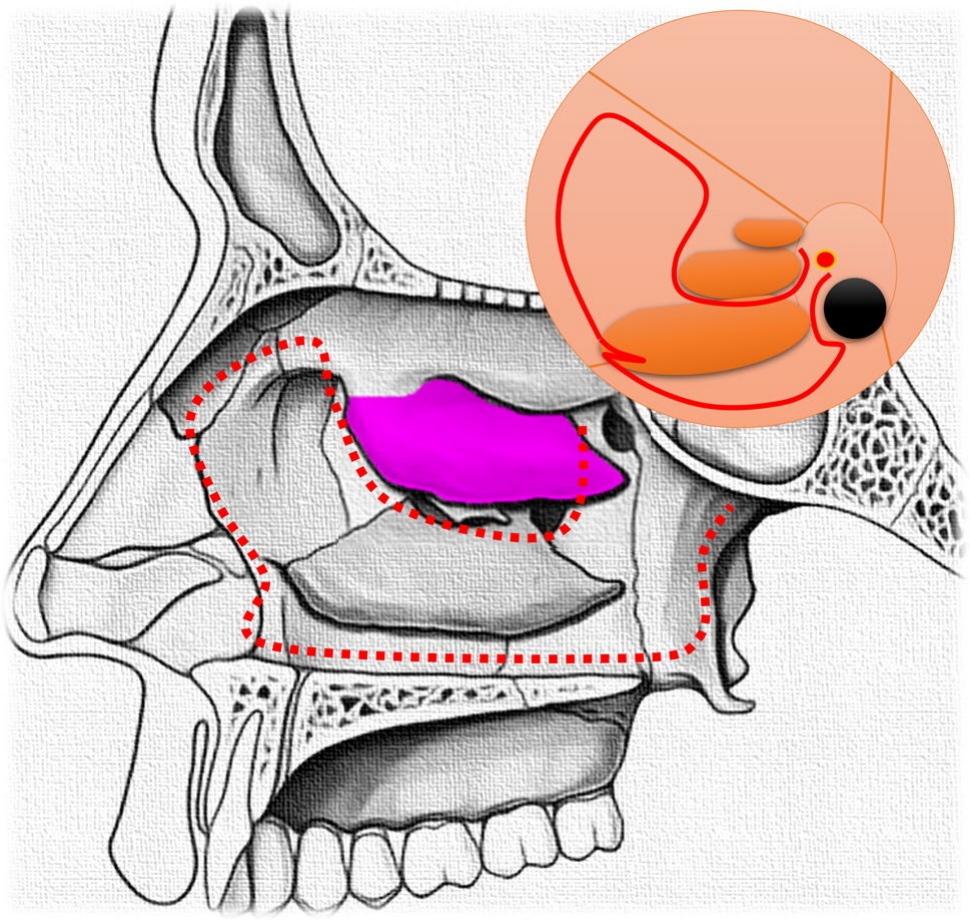
The border of the incisions for the right LNWF

The posterior pedicled LNWF can be performed on either the left side or the right side, although the one with a robust inferior turbinate would be preferred. The margin of the flap can be simply divided into three groups of incisions: anterior, superior, and inferior. The border of the incisions, which should to be made deep into the submucoperiosteal plane using a needle-tipped monopolar or coblator, is defined by bony landmarks (Fig. 1).

### Inferior incision (Fig. 2)

The inferior incision may be tailored to many variations depending on the needs of each case. To harvest a wider surface flap, the incision may extend inferiorly and medially along the nasal floor; thus, LNWF can also be expanded in combination with the nasal floor and residual septal flap. Posteriorly crossing the horizontal plate of palatine bone, the incision would be extended backward along the medial pterygoid plate over the superior margin of the eustachian tube, making room for pedicle rotation.

**Fig. 2:**
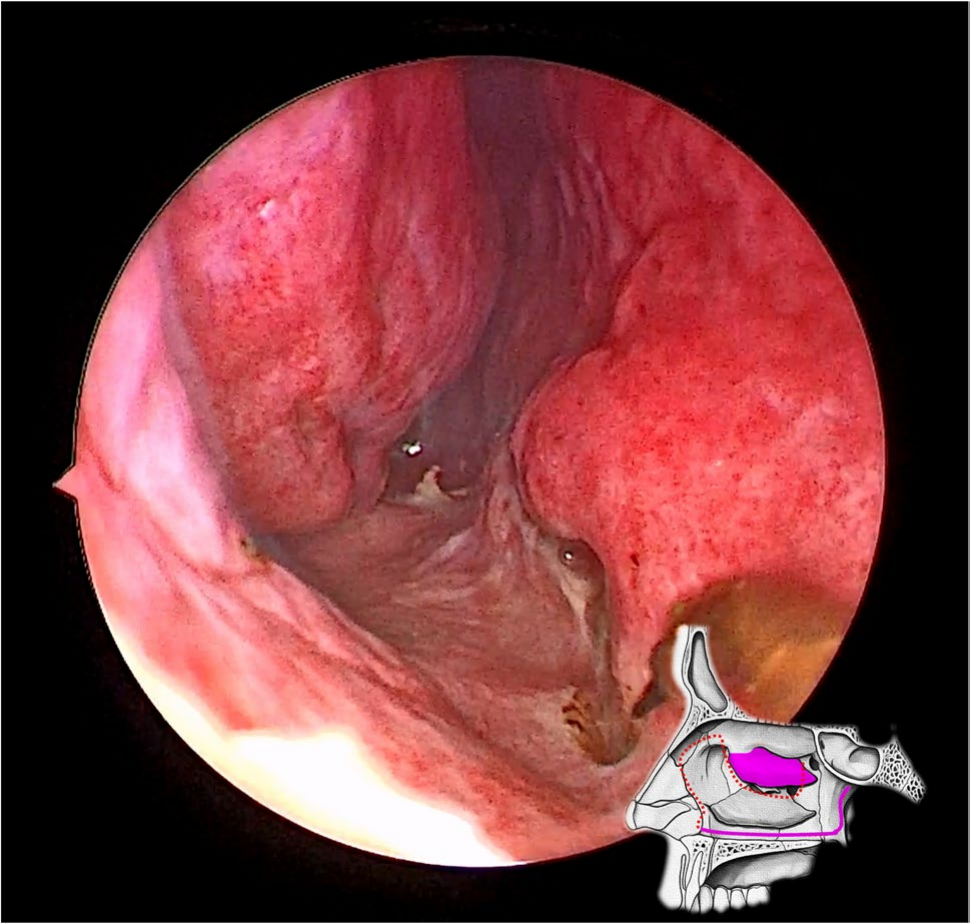
Inferior incision of the right LNWF

### Anterior incision (Fig. 3)

The mucoperiosteum was cut along the coronal plane at the junction between the skin and mucosa of the medial rim of the piriform aperture. Crossing through the head of the inferior turbinate, the incision then extends upward along the frontal process of the maxilla to the nasal roof.

**Fig. 3:**
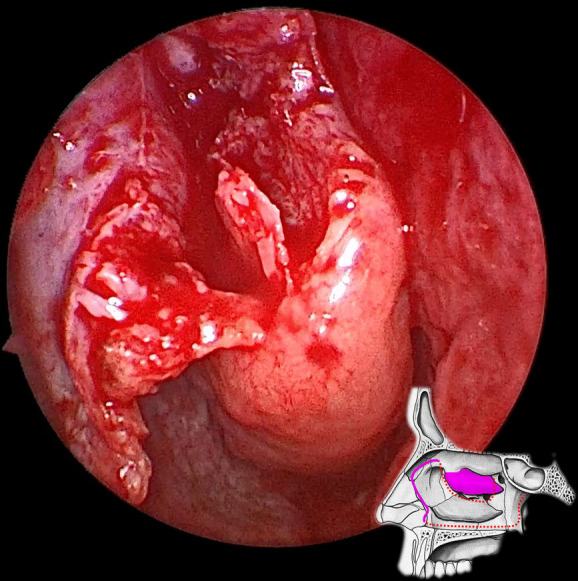
Anterior incision of the right LNWF

### Superior incision

The incision first extends along the uppermost nasal roof to the agger nasi, and then it descends along the posterior aspect of the lacrimal bone to the axilla of the middle turbinate. This is done to harvest the upper and anterior mucosa of the nasal lateral wall, which contributes to the main difference from the inferior turbinate flap. The mucosa from the superior surface of the ethmoid crest to the orbital process of the palatine bone might be incised during the following submucosal steps.

**Fig. 4:**
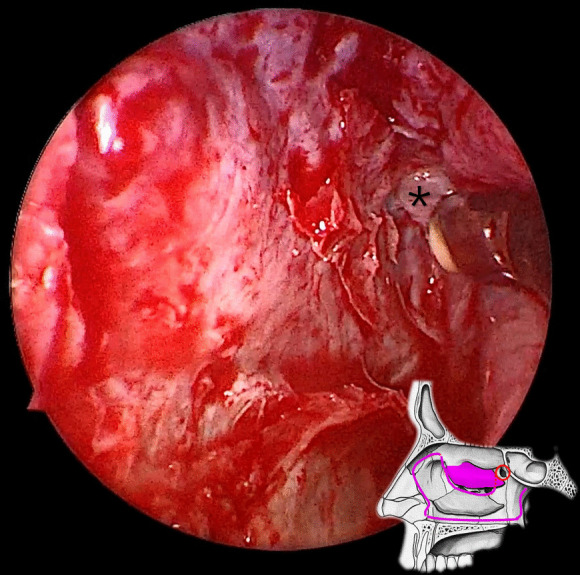
Elevation of the right LNWF. *: sphenopalatine foramen

- 2. Elevation of the flap (Fig. 4)

An incision is made along the contour of the head of the inferior turbinate, with the length just enough for the LNWF mucosa to unfold like a book. Two main structures will hinder the flap elevation: one is the bone at the head of the inferior turbinate; the other is the nasolacrimal duct. After piecemeally remove the inferior turbinate bone in front of the nasolacrimal duct, the opening of the duct should be sharply transected and concurrent dacryocystorhinostomy may be performed. While the flap continuously elevated anteriorly to posteriorly, the bone of the inferior turbinate is fractured and removed, noting that the densely adherent bone at the root of the inferior turbinate may contain branches of the inferior turbinate artery, and it should not be forcibly extracted.

- 3. Free and rotation of the pedicle

**Fig. 5:**
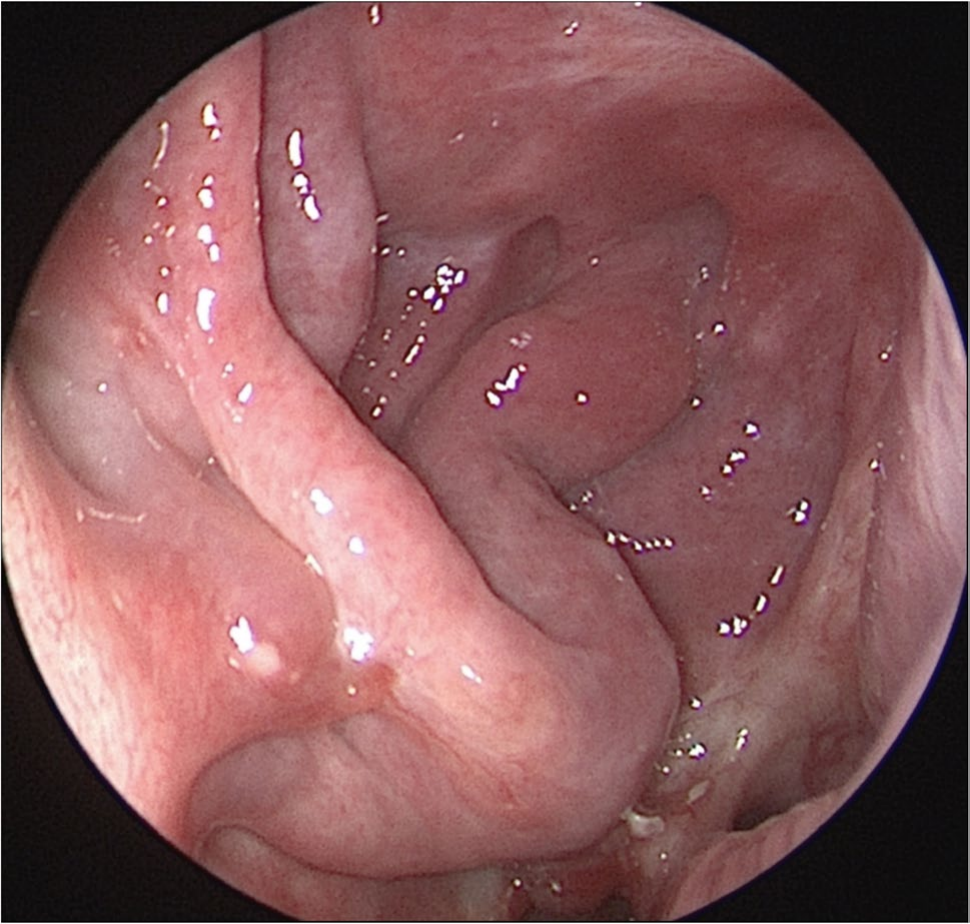
Follow-up endoscopy after the right LNWF reconstruction

Once fully creating the subperiosteal space, dissection should be directed toward the sphenopalatine foramen, which should be identified beforehand by the ethmoid crest of the palatine bone. When inferiorly to superiorly elevating the mucosa overlying the posterior middle meatus, any encountered branch of PLNA should be carefully preserved following strictly subperiosteal dissection. Removal of the anterior and posterior walls of the pterygopalatine fossa and releasing the vascular pedicle facilitates the rotation and extension of the flap while taking the risk of pedicle injury. After completing the aforementioned steps, the flap can be unfolded and rotated posteriorly 90 degrees to cover the clival defect or 180 degrees for the defect of the sellar region (Fig. 5).

### Indications

1. Bilateral HBF is not available in the following situations [3, 4, 9]:
  a The HBF pedicle is compromised by prior nasal septum operation, septal perforation, or previous surgeries such as wide sphenoidotomy and posterior septectomy.
  b The HBF pedicle or posterior septal mucosa is involved by tumor or pathology.
  c The posterior nasal septal artery was injured during the operation.
2. Large skull base defects result in high-flow CSF leak, or they cause ICA and other vital vascular or nerve tissue to be exposed in the following locations [2]:
  a Clivus
  b Nasopharynx
  c Sella and parasella area
  d Planum sphenoidale
  e Posterior obit

## Limitations

1. Similarly to HBF, LNWF is not appropriate for situations in which the inferior turbinate has atrophy from prior surgery or radiation therapy, or if the vascular pedicle has been compromised by lesions.
2. Compared with HBF, the posterior pedicled LNWF is not amenable to obtaining sufficient length to cover the entire anterior cranial fossa due to the limited arc of rotation and finite extension [5].

## How to avoid complications

- 1. Complications of the flap donor site

Dissection of the nasal mucosa may cause nasal morbidity such as epistaxis, hyposmia, and crusting; thus, nasal irrigation and endoscopic debridement would be helpful. Although empty nose syndrome could theoretically result from inferior turbinate excision, this complication was not observed in the follow-up of our cases. Transient epiphora due to operating around the outflow of the nasolacrimal duct was found in 1 case and resolved by lacrimal irrigation subsequently.

- 2. Complications of the flap recipient site

Tearing of the mucosa during dissection may lead to failure of repair, and multi-layer reconstruction and postoperative lumbar drainage can reduce the risk. In addition, skeletonizing the sphenoid sinus as well as removing the nonvascularized tissue surrounding the defect is another key to effective reconstruction in revision surgery.

### Specific information for the patient

In addition to taking into account the above-mentioned complications, patients should be informed of other repair options from intranasal [1] and extranasal regional flaps [6, 8].

## 10 key points summaries

1. Pedicled nasal flaps can provide reliable skull base reconstruction.
2. Secondary surgical intervention makes HBF harvesting infeasible or unreliable, rendering skull base reconstruction challenging in such scenarios.
3. When HBF is not available, LNWF emerges as an achievable and practical alternative.
4. LNWF is an extension of the inferior turbinate flap to the lateral nasal wall, which can further extend towards the nasal floor, forming an even larger mucoperiosteal flap.
5. LNWF is particularly suitable for the repair of clivus, and can also be utilized for the reconstruction of sellar or the supersellar region.
6. Harvesting LNWF requires an adequate comprehension of the anatomy of the posterior lateral nasal artery and the lateral nasal wall.
7. The dissection of LNWF is more challenging than that of HBF, calling for a step-by-step approach directed by experienced technical nuances.
8. In addition to the postoperative nasal morbidity, LNWF theoretically carries the risk of empty nose syndrome and epiphora, while in reality, complications are minimal.
9. The posterior pedicled LNWF is unable to repair the defect of the anterior skull base adjacent to the frontal sinus, necessitating the application of alternative techniques.
10. Destruction of vascular supply and atrophy of the inferior turbinate preclude the use of LNWF.

## Supplementary Information

Below is the link to the electronic supplementary material.

ESM 1 Supplementary Material 1 (MOV 190 MB)

## Data Availability

The data and materials supporting this study are available from the corresponding author upon reasonable request.

## Abbreviations

CSF: Cerebrospinal fluid
HBF: Hadad-Bassagasteguy flap
LNWF: Lateral nasal wall flap
PLNA: Posterior lateral nasal artery
